# Leptospirosis in Unconventional Mammal Pets

**DOI:** 10.3390/vetsci12030285

**Published:** 2025-03-19

**Authors:** Fabrizio Bertelloni, Valentina Virginia Ebani

**Affiliations:** 1 Department of Veterinary Sciences, University of Pisa, Viale delle Piagge 2, 56124 Pisa, Italy; fabrizio.bertelloni@unipi.it; 2 Centre for Climate Change Impact, University of Pisa, Via del Borghetto 80, 56124 Pisa, Italy

**Keywords:** leptospirosis, unconventional pets, diagnosis, prophylaxis, public health, zoonosis

## Abstract

The demand for unconventional pets has markedly increased in recent years worldwide. Among them, many species of mammals are frequently kept in domestic environments in close contact with their owners. Pets often harbor zoonotic microorganisms without showing clinical signs; therefore, owners do not suspect that they can be a source of pathogens. Pets of several unconventional species may act mainly as maintenance hosts for leptospires; they are clinically silent but shed the spirochetes in their urine representing a serious risk of infection for people living in the same domestic area. However, their role as maintenance or incidental hosts seems variable in relation to the animal species, and it has not always been elucidated. No vaccines against *Leptospira* spp. are available for unconventional mammal pets, and so prophylaxis is based on rigorous hygienic measures and the identification of infected animals through indirect and/or direct diagnosis for leptospirosis.

## Simple Summary

Unconventional mammal pets are largely present in domestic environments where they can act as a source of zoonotic agents. They often have no clinical signs; therefore, owners do not suspect any risk of infection. Among the zoonotic pathogens, *Leptospira* spp. is one of the most dangerous pathogens for humans, but unconventional pets, such as rodents, rabbits, mustelids, and others, are rarely submitted to diagnosis to evaluate if they harbor leptospires. Lack of information about the epidemiological role of most of these animals as maintenance or incidental hosts contributes to making leptospirosis in unconventional mammal pets a poorly known concern.

## 1. Introduction

A companion animal has been defined as “*any domesticated, domestic-bred or wild-caught animals, permanently living in a community and kept by people for company, enjoyment, work (e.g., support for blind or deaf people, police or military dogs) or psychological support—including, but not limited to dogs, cats, horses, rabbits, ferrets, guinea pigs, reptiles, birds, and ornamental fish.*” [1].

The American Society for the Prevention of Cruelty to Animals states that companion animals should be domesticated or domestically bred animals whose physical, emotional, behavioral, and social needs can be readily met as companions in the home or in close daily relationships with humans [2]. Companion animals can be dubbed with different names, such as pet and family animal [3]. CALLISTO (Companion Animals multisectoriaL Interdisciplinary Strategic Think tank On zoonoses) defines companion animals as ‘any domesticated, domestic-bred or wild-caught animal permanently living in a community and kept by people for company, amusement, work (e.g., support for blind or deaf people, police or military dogs) or psychological support including dogs, cats, horses, rabbits, ferrets, guinea pigs, reptiles, birds and ornamental fish’ [4]. The British Small Animal Veterinary Association (BSAVA) considers non-traditional (unconventional) companion animals as those animals that are not traditionally domesticated or kept as pets and whose welfare needs can be more difficult to meet in a domestic environment [5].

The large proliferation of traditional and non-traditional companion animals has increased the risk of transmission of zoonotic microorganisms to their owners. In fact, some pathogens, usually related to conventional animal species, can also be harbored and transmitted by less known animals.

Canine leptospirosis is a widely investigated infectious disease, and the risk of transmission of leptospires from infected dogs to people is well known [6]. The disease in dogs may be present in two different forms: Weil’s Disease, mainly caused by the serovars Icterohaemorrhagiae and Copenhageni, and Typhus Disease or Stoccarda Disease, mainly related to the serovars Canicola and Bratislava [7]. Human leptospirosis is commonly known as Weil’s Disease, even though serovars other than Icterohaemorrhagiae can cause severe clinical signs [7].

Leptospires may infect other mammals, including small species such as rodents and rabbits, as well as cold-blooded animals that are frequently kept in domestic environments as pets. Usually, these animals do not develop clinical signs even if they are infected by leptospires; they can act as maintenance hosts which shed the spirochetes within their urine. For this reason, owners of unconventional pets must be informed about the possible risk of being infected by leptospires shed by their animals. Diagnosis and prophylaxis are pivotal in reducing the risk of transmission from animals to humans, but they are not commonly performed since veterinarians and owners rarely associate leptospirosis with these animal species. Although many unconventional species are considered susceptible to *Leptospira* infection, specific studies about its occurrence, epidemiology, and pathological characteristics are very scarce.

The aim of the present narrative review was to collect data about *Leptospira* infection in unconventional mammal species, often kept as companion animals, with particular emphasis on the role they have as potential sources of infection for owners, either as maintenance or incidental hosts.

## 2. Leptospira

### 2.1. Etiology

The genus *Leptospira*, family Leptospiraceae, order *Spirochaetales*, includes Gram-negative spirochetal bacteria. Recent genomic analyses have led to a significant revision of *Leptospira* taxonomy. The genus now encompasses over 60 species categorized into two clades (P and S) and further divided into four subclades (P1, P2, S1, and S2). The P1 subclade includes pathogenic species, while P2 comprises strains with intermediate pathogenicity. In contrast, the saprophytic strains are found in subclades S1 and S2 [8].

Strains within the same species can be differentiated into serovars based on traditional antigenic differences or through more recent molecular techniques, such as Multi-Locus Sequence Typing (MLST). Serovars that exhibit antigenic similarities are grouped in serogroups [9,10].

Leptospires have a distinctive helical shape, a length of 6–20 µm, a diameter of 0.1 µm, and a wavelength of 0.5 µm. Their ends are similar to a hook, and their motility is attributed to the presence of two periplasmic flagella inserted at the poles [11].

*Leptospira*’s virulence is related in large part to the lipopolysaccharide (LPS) of the bacterial wall, which is the main antigen during infection and is responsible for antigenic diversity and serogroup classification [12].

When pathogenic *Leptospira* (P1) are excreted through the urine of infected animals, they immediately need to be in a humid environment or body of water to survive. However, the presence of pathogenic leptospires in the soil has been demonstrated. Pathogenic *Leptospira* usually survive in soil with a humidity of <20%, but they can survive in wet soil during dry days and emerge to the surface during rainy days. Leptospires lasted for three days in natural seawater and only survived 12 h in sodium chloride [13]. Moreover, it has been demonstrated that pathogenic leptospires may survive at 4 °C for 130 days, at 20 °C for 236 days, and at 30 °C for 316 days, and, based on pH levels, they survive at pH 7 for 344 days and at pH < 7 for 129 days. In all cases, pathogenic leptospires cannot multiply in the environment [14,15].

### 2.2. Epidemiology

Pathogenic species have the capacity to infect a wide range of animal species, including humans [11,16]. Animals can either act as non-clinical carriers of leptospires or exhibit symptoms of infection. Traditionally, *Leptospira* hosts are categorized into maintenance hosts and incidental hosts. Maintenance hosts harbor the spirochetes primarily within their kidneys although they do not exhibit disease symptoms or do so infrequently. These hosts play a crucial role in the dissemination of leptospires by shedding them in their urine. Different animal species serve as maintenance hosts for different *Leptospira* serovars. Conversely, incidental hosts encompass any animal species, including humans, that may develop clinical signs upon infection. Incidental hosts contribute less to the transmission of *Leptospira* compared to reservoirs, as their bacterial excretion is generally limited. Furthermore, an animal species may act as a reservoir for a specific serovar while serving as an incidental host for others [17,18].

Leptospires are primarily expelled by infected animals through their urine, with susceptible hosts becoming infected either through direct contact with infected animals or indirect exposure to contaminated material. Infection can occur via mucous membranes of the eyes, mouth, nose, and genital tract, as well as through injured skin [18]. Venereal and vertical transmission have also been documented in some mammal species [18,19]. It is plausible that leptospiral infection in unconventional mammal pets occurs through the same routes documented in other animal species, although specific studies about venereal and vertical transmission have not been conducted. Their role as maintenance or incidental hosts is varies depending on the species and has not always been clearly elucidated.

## 3. Companion Animals

Many small mammals are kept as household pets, including mice, rats, hamsters, guinea pigs, gerbils, chinchillas, and degus. These species are commonly owned in several parts of the world. However, some small mammal species may not be permitted in apartments or rental homes, and certain species are illegal as pets in some regions due to their classification as invasive animals [20].

Since the beginning of the last century, some small rodent species has been well known for their susceptibility to leptospires and have been extensively employed in laboratory research. Animals such as hamsters and guinea pigs have played a significant role in the production of sera containing anti-*Leptospira* antibodies. They have also been inoculated with tissue or blood samples from animals suspected to be infected by leptospires for direct diagnostic purpose [21]. Despite their known susceptibility of these animals to leptospires, information about the occurrence and pathological characteristics of leptospirosis in most unconventional pet species remain scarce.

### 3.1. Rodents

#### 3.1.1. Hamster

Various hamster species belong to the family Cricetidae within the order *Rodentia*. Among them, *Mesocricetus auratus*, commonly known as the golden hamster or Syrian hamster, is one of the most frequently used model organisms for the study of leptospirosis. *Mesocricetus auratus* is also popular as a house pet due to its docile nature, cuteness, and small size. Other commonly kept hamster species include Campbell’s dwarf Russian hamster (*Phodopus campbelli*), Roborovski dwarf hamster (*Phodopus roborovski*), dwarf winter white Russian (*Phodopus sungorus*), European hamster (*Cricetus cricetus*), and Chinese hamster (*Cricetus griseus*) [22], but no data about the occurrence of leptospirosis in these companion animals are available, except for a recent survey by Ulsenheimer et al., who found *L. interrogans* DNA in the kidney of one pet *M. auratus* [23].

Usually, hamsters are considered highly susceptible to a wide variety of pathogenic *Leptospira* strains and are therefore widely used as animal models for studying acute leptospirosis [24]. Their high sensitivity to leptospires classifies them as incidental hosts.

#### 3.1.2. Guinea Pig

Young guinea pigs (*Cavia porcellus*; order *Rodentia*, family Caviidae) are widely used as experimental models for acute leptospirosis. Typically, guinea pigs are infected through the intraperitoneal inoculation route, which induces a lethal infection characterized by clinical signs comparable to those observed in severe human leptospirosis cases. Experimental infection of guinea pigs through epicutaneous inoculation has also been studied; this route, which better reflects natural infection, induces symptoms and pathological lesions similar to those observed after intraperitoneal infection [25]. In all cases, guinea pigs have been shown to be susceptible to leptospires and seem to act as incidental hosts.

Conversely, some studies suggest that guinea pigs may also serve as possible maintenance hosts. In fact, antibodies to *Leptospira* spp. were detected in 40.50% (98/242) of apparently healthy guinea pigs from five family-run commercial breeding farms in Cajamarca, Peru [26]. In particular, the reactive serovars identified were Icterohaemorrhagiae (19.01%, 46/242), Canicola (16.53%, 40/242), and Pomona (8.68%, 21/144), with antibody titres ranging from 1:100 to 1:200. In addition, a survey conducted in clinically healthy guinea pigs raised for human consumption in Colombia detected *L. interrogans* sensu stricto in 1.85% (5/270) of the analyzed animals; in particular, *Leptospira* DNA was identified in the kidneys of four animals using PCR, while in one case, spirochetes were detected via the Warthin–Starry staining [27].

Baby guinea pigs have been shown to be susceptible to *Leptospira* infection but develop resistance as they mature. Increased resistance with age and weight gain of guinea pigs has been correlated with the production of agglutinating antibodies to leptospires and the maturation of B-cell-dependent (but not T-cell-dependent) areas in lymphoid organs [28]. Thus, the epidemiological role of guinea pigs may vary depending on the animal’s age as well as the involved serovar.

#### 3.1.3. Rat and Mouse

Rats are found throughout the order Rodentia, family Muridae, but stereotypical rats are found in the genus *Rattus* which includes different species. Other rat genera include *Neotoma* (pack rats), *Bandicota* (bandicoot rats), and *Dipodomys* (kangaroo rats) [29]. Mice are rodents smaller than rats. The best-known species is the common house mouse (*Mus musculus*), but other species encompassed in the family Muridae are known [30]. Rats and mice make up approximately 95% of all laboratory animals used in biomedical research [31].

Rats and mice are commonly known as maintenance hosts for different serovars, mainly Icterohaemorrhagiae [32]. Despite this, the number of owners keeping these animals as pets in their houses is constantly increasing [33].

Information about leptospirosis diseases in pet rats is lacking in the literature, but outbreaks of infection have been observed in laboratory rats [34], as well as laboratory rats have been used in model infections with serogroup Icterohaemorrhagiae [35].

Brockmann et al. [36] carried out an epidemiological study in Germany to evaluate the risk factors associated with human leptospirosis; the contact with animals was confirmed as a fundamental factor, and the involvement of pet rodents, in particular rats, mice, and guinea pigs was found to be the most relevant. Confirming these observations, the scientific literature reports some cases of human leptospirosis associated with pet rats. A case of human leptospirosis, caused by the serovar Icterohaemorrhagiae and acquired from pet rats purchased in a pet shop was reported by Gaudie et al. in 2008 [37] in the United Kingdom. Similarly, white fancy rats (domestic albino *Rattus norvegicus*) were identified as the potential infection source for acute leptospirosis in a human immunodeficiency virus (HIV)-positive patient in Germany. *Leptospira interrogans* serovars Icterohaemorrhagiae or Canicola were identified as the etiologic agent of the human infection, and the same agent was found in the kidneys of the euthanized rats owned by the patient [38]. A case of *L. interrogans* serogroup Icterohaemorrhagiae infection in a young woman was associated with the adoption of a semi-wild rat in the United Kingdom [39]. Similarly, in Sweden, a human case of Weil’s Disease was reported. The patient revealed that he owned four white pet rats; after euthanizing the animals, blood and kidney samples were taken, and serovars Icterohaemorrhagiae or Copenhageni were cultured from all kidney samples [40].

In addition, a case of leptospirosis meningitis in a previously healthy young woman who was most likely infected by her pet mouse was reported in Denmark. The patient had no recent travel history but kept mice as pets, and one of them had fallen ill with conjunctivitis 1.5 months prior to the onset of the patient’s symptoms [41].

The cases of leptospirosis in three patients with various severities of the disease occurred between 2005 and 2010 in the Czech Republic; all cases were related to the pet rats owned by the patients [42].

Cases of leptospirosis in patients owning pet rats or mice were also documented in Belgium and France between 2009 and 2016 [43]. Owners had severe disease with typical symptoms and serological diagnosis detected infections by serogroup Icterohaemorrhagiae or Sejroe. Their pet rodents were euthanized after obtaining the approval from the owner and urine and kidneys were collected for analyses; *Leptospira* spp. DNA was found with PCR, whereas bacteriological cultures allowed to identify the serogroups Icterohaemorrhagiae and Sejroe. All patients were regularly exposed to their pet rodents and the environments where the animals lived; in fact, they declared that their pet rodents were usually kept in cages but also occasionally left to freely roam in the household. One patient used mice for magic tricks in nurseries while another one worked in a pet shop, owned eleven pet rats, and volunteered in a pet-rat daycare association. In other cases, the patients kept pet mice or rats at home [43].

#### 3.1.4. Gerbil

Gerbils (*Meriones* spp.; order *Rodentia*, family Muridae) are known as animals susceptible to leptospirosis and for this reason they have been largely used in laboratory research for studying *Leptospira* spp. In particular, the gerbil has been identified as a good animal model to study pulmonary hemorrhage as a consequence of severe leptospirosis infection. Moreover, the gerbil mimics the human in its response to the infection by producing increased amounts of platelet-activating factor acetylhydrolase [44,45].

In 1976, Tripathy and Hanson [46] observed leptospiruria with persistent microscopic agglutinating antibody titers in gerbils that became carriers following experimental infection with the serovar Grippotyphosa and leptospires were isolated from the kidneys of a gerbil that died 28 months after an experimental inoculation. Although no clinical signs of disease were shown by the animals, the researchers hypothesized that death was due to chronic leptospiral infection as observed by gross and histopathologic changes. The persistence of a prolonged carrier state has a relevant epidemiological significance, but it does not mean that gerbils are maintenance hosts. In fact, Van der Hoeden [47] (1954) suggested that leptospirosis in gerbils is self-limiting in nature because these animals succumb soon after infection. This observation was confirmed by the findings of Yukawa et al. [48] (1990) which proved that the Mongolian gerbil (*Meriones unguiculatus*) is highly susceptible to *L. interrogans* serovars such as Icterohaemorrhagiae, Copenhageni, Canicola, Autumnalis, Javanica, Pyrogenes, and Hebdomadis [48]. In fact, gerbils died after experimental intraperitoneal inoculation with these serovars, and hemorrhage and jaundice, common lesions in incidental hosts, were observed [48].

#### 3.1.5. Degu

The degu (*Octodon degus*; order *Rodentia*, family Octodontidae) is a medium-sized rodent belonging to the suborder *Hystricomorpha*, along with the guinea pig and chinchilla, and it is native to mountainous areas of Chile where it is currently endemic. Degus are commonly used as laboratory animals, mainly as models of human disease, and, over the past few years, they have become increasingly popular as pets [49,50].

Natural infections by pathogenic *Leptospira* in free ranging degus (*O. degus*) and Darwin’s pericotes (*Phyllotis darwini*), another rodent species, were reported by Correa et al. [51]. *Leptospira* DNA was detected in kidney and urine samples of 26/260 (10%) of the degus captured and analyzed in different years and sites in Chile. In addition, anti-*Leptospira* antibodies were detected; in particular, sera were tested with the microscopic agglutination test (MAT) using a panel of 15 serovars representing 11 serogroups and five species (*Leptospira biflexa*, *Leptospira borgpetersenii*, *Leptospira interrogans*, *Leptospira kirschneri*, and *Leptospira santarosai*). Only two animals were seropositive, with 1:50 antibody titer for the serovars Bratislava (*L. interrogans*) and Ballum (*L. borgpetersenii*).

The social behavior of degus living in natural conditions could favor the transmission of *Leptospira* both directly and indirectly, because they form groups with cooperative breeding [52]. No data about *Leptospira* infection in pet degus is available; however, the survey of Correa et al. [51] proved the susceptibility of these animals to the spirochetal pathogens and suggested these animals as possible maintenance hosts.

#### 3.1.6. Chinchilla

The long-tailed chinchilla (*Chinchilla lanigera*; order *Rodentia*, family Chinchillidae), also called the Chilean, coastal, common, or lesser chinchilla, and the short-tailed chinchilla (*Chinchilla chinchilla*), are endangered species in the wild after historically being hunted for their soft hair coats. Domestic breeds of chinchilla are believed to descend from *C. lanigera*. Today, chinchillas are successfully bred in captivity for biomedical research as well as for their fur and the pet trade.

Turner in 1961 [53] experimentally infected twenty chinchillas (*Chinchilla lanigera*) with a field strain of the serovar Pomona through intraperitoneal and intraocular inoculation. All animals were susceptible to the infection: leptospires were isolated from blood and chinchillas developed lesions of the liver, kidneys, lungs, and spleen.

Even though the information about *Leptospira* infection in captive chinchillas are almost absent, some cases of nephritis in captive *C. lanigera* have been observed and attributed to the serovars Icterohaemorrhagiae and Pomona in Argentina [54]. The high susceptibility of chinchillas to *Leptospira* infection suggests these animals as incidental hosts.

### 3.2. Glider

The sugar glider (*Petaurus breviceps*; order *Diprotodontia*, family Petauridae) is a small omnivorous, arboreal, and nocturnal marsupial gliding possum; it is native to Australia, but it has become popular as exotic pet in other continents [55].

Data on the occurrence of leptospirosis and the characteristics of the disease in gliders are not present in the scientific literature, but sugar gliders are considered to be susceptible to this pathogen. The infected animals can be carriers of leptospires without showing clinical signs, although the infection can cause liver and kidney disorders [56]. However, the limited data available on leptospirosis in gliders makes it difficult to classify these animals as maintenance or incidental hosts.

A case of leptospirosis in a woman hospitalized in Rhode Island (USA) was suspected to be related to a sugar glider; the patient worked in a pet shop where she was bitten on the hand by a *P. breviceps* 4 days prior to the hospital admission. However, the patient also frequently attended livestock auctions where she handled farm animals [57].

### 3.3. Hedgehog

Hedgehogs are frequently kept as pets, as well, although in many countries it is illegal [58]. They are small, nocturnal, spiny-coated insectivores classified into several species of the family Erinaceidae, included in the order *Eulipotyphla*, but the European hedgehog (*Erinaceus europaeus*) and the smaller African pygmy hedgehog (*Atelerix albiventris*) are more commonly seen as companion animals [58].

Pet hedgehogs have been identified as sources of different zoonotic pathogens for humans [58], because often they do not develop clinical signs therefore owners do not suspect they can be infected and must be treated. *Leptospira* spp., mainly serovars Australis/Bratislava, Ballum, and Canicola often infect wild hedgehogs which are considered as important maintenance hosts [21,59,60,61,62,63]. In view of the susceptibility of these animals to leptospires, it is supposable that pet hedgehogs might harbor the spirochaetes in their kidneys without developing clinical signs and contaminate domestic environment with their infected urine.

### 3.4. Mustelids

The family Mustelidae is the largest family within the order Carnivora and includes many species such as stoats, polecats, mink, fishers, wolverines, weasels, martens, badgers, and otters. Usually, mustelids are free ranging in terrestrial and marine environments, but some species are used as laboratory animals, managed in zoological settings, farmed and kept as pets [64]. Ferrets (*Mustela putorius furo*) are the most common pet mustelids. Data about leptospirosis in mustelids is scarce, although these animals are considered susceptible to leptospiral infection. Lacomb et al. [65], in France, reported a case of human leptospirosis whose highly suspected vector was a domestic ferret. The affected man owned two domestic ferrets, which he used for hunting in previous years when they had been in contact with rodents. The ferrets, which seem to act as maintenance hosts, shed leptospires in their urine without showing clinical signs. This case highlighted the difficulty identifying the origin of the infection in some cases of human leptospirosis. In fact, only after several days of the hospitalization, the diagnosis for leptospirosis was requested for the patient and only after an accurate anamnesis was the infection associated with domestic ferrets; moreover, the veterinarian who examined the animals twice failed to collect their urine to prove the chronic kidney infection.

A serological investigation carried out in free-ranging and farmed mustelids from southwestern France detected high prevalences: 73.7% (73/99) in European mink (*Mustela lutreola*), 65.4% (87/133) in European polecats (*Mustela putorius*), 86.4% (64/74) in American mink (*Mustela vison*), 89.4% (17/19) in stone martens (*Martes foina*), 73.7% (14/19) in pine martens (*Martes martes*), and 31.4% (16/51) in farmed American mink [66]. In addition, molecular analyses found renal carriage in 23% (8/34) European minks, 22% (4/18) polecats, and 15% (5/33) free-ranging American mink. However, the researchers supposed that these wild mammals have not a relevant epidemiological role, because they found that most seropositive animals were PCR-negative, suggesting a short urinary excretion [66].

*Leptospira borgpetersenii* was recently found in a ferret (*M. putorius furo*) by Wilkinson et al. [67], who in this way detected new host-*Leptospira* associations in wildlife in New Zealand, also including stoats and brushtail possums infected with the serovar Copenhageni. The role of mustelids in the epidemiology of leptospirosis probably depends on the animal species. For example, striped skunk (*Mephitis mephitis*) has been supposed to be reservoirs by Shearer et al. [68], who found in Ontario, Canada, 42% of skunk kidneys positive for *Leptospira* on immunohistochemistry. Globally, mustelids have been known to harbor *Leptospira* from serogroups Icterohaemorragiae, Australis, Autumnalis, Grippotyphosa, and Sejroe [61].

### 3.5. Rabbit

Rabbits belong to family Leporidae included in the order *Lagomorpha*, which encompasses several genera among which *Oryctolagus* and *Sylvilagus* are the most widespread. The former, *Oryctolagus*, includes the European rabbit (*Oryctolagus cuniculus*) which is considered the ancestor of hundreds of breeds of domestic rabbits [69]. Rabbits are commonly kept as pets as well as employed as research subjects for different purposes, including the study of leptospirosis [70].

The role of rabbits as carriers of *Leptospira* has been proven [71]. Previous studies found variable *Leptospira* seroprevalence rates in rabbits’ population, depending on the test used, the number of animals and geographic area of provenience [6].

Shotts et al. [72] analyzed for leptospirosis 50 free-ranging rabbits, 44 cottontails (*Sylvilagus floridanus*), and 6 swamp rabbits (*Sylvilagus aquaticus*) from the Mississippi Delta Area (USA). *Leptospira interrogans* antibodies were demonstrated in 77% (37/48) of the sera collected; the serovars most frequently encountered were Ballum, Australis, Icterohaemorrhagiae, Canicola, and Grippotyphosa. When organs were examined, focal nephritis was observed histologically in 92% (46/50) of the kidneys. In addition, Grippotyphosa was cultured from 8% (4/50) of the kidneys collected [72]. An interesting serological survey carried out in companion rabbits in Iran found 9/68 (27.94%) animals positive for the serovars Icterohaemorrhagiae, Tarassovi, Grippotyphosa, Hardjo, Pomona, and Australis. At the time of blood collection, all investigated animals seemed to be healthy, and no clinical signs of leptospirosis were observed [73]. These findings highlighted the potential role of *Leptospira*-infected rabbits, including pets, as maintenance hosts, which after contracting leptospires in breeding facilities, pet shops, or house gardens can develop a chronic infection and become a source of leptospires for months or years [6], for owners and other animals living in domestic environments where often they roam freely.

The importance of pet rabbits as a potential source of leptospires has been recently shown by Ulsenheimer et al. [23], who tested pets belonging to different unconventional species, including rabbits and DNA of *L. interrogans* was detected in 3/23 (13.04%) rabbits (*O. cuniculus*).

### 3.6. Minipig

Minipigs (*Sus scrofa domestica*; order *Artiodactyla*, family Suidae), also called miniature pigs, are more and more frequently kept in domestic environments as pets; minipigs are employed in pet therapy as well. Furthermore, some breeds are widely used in biomedical research [74]. Usually, they belong to breeds such as the Vietnamese pot-bellied, which are smaller than farm pigs. However, since pigs can breed years before they fully mature, unscrupulous or ignorant breeders may show off parent pigs which are not fully grown themselves and have not reached their full adult size.

Studies about the occurrence and main characteristics of leptospirosis in this animal category are not available, but it is well known that pigs are susceptible to *Leptospira* spp. acting as both maintenance and incidental host in relation to the involved serovar. Swine are maintenance hosts, harboring leptospires in kidneys and genital tract, for many serovars including Canicola, Grippotyphosa, Icterohaemorrhagiae, and Pomona. Swine disease is mainly caused by the serovars Australis, Canicola, Grippotyphosa, Hardjo, Icterohaemorrhagiae, Pomona, and Tarassovi, which cause infertility, abortion, neonatal death and weakness [75].

Studies about leptospirosis in pet minipigs are not available in the literature. However, a study proved the susceptibility of miniature pigs on a par with what occurs in farm pigs. In fact, Sakoda et al. [76] experimentally infected, through intraperitoneal inoculation, four minipigs with *L. interrogans* serovar Manilae to evaluate its pathogenicity. Leptospires were recovered from the whole blood, kidneys, and livers in the acute phase without showing any clinical signs. Under immunosuppressive conditions by dexamethasone, leptospires were recovered from the kidneys and their genes were detected from the urine in the chronic phase. It is supposable that miniature pigs strictly kept in domestic areas have reduced occasion to acquire leptospires from the environments; however, when they spend time outdoors, mainly if owners live in peri-urban and rural zones, they can have contact with urine of *Leptospira*-infected wild animals, mainly rodents and hedgehogs; in relation to the involved serovar, minipigs may act as maintenance hosts which do not show any symptoms or incidental hosts with clinical forms of variable severity.

The cases of human leptospirosis reported in the literature and associated with infected unconventional pets are summarized in Table 1.

On the basis of the information collected from the literature, Table 2 reports the epidemiological role of unconventional mammal pets in the epidemiology of leptospirosis.

## 4. Diagnosis

In humans and traditional domestic animals, laboratory diagnosis of leptospirosis primarily relies on serological tests. Although anti-*Leptospira* antibodies typically develop about a week after the onset of symptoms, serological methods can deliver reliable results relatively quickly [77,78]. The microscopic agglutination test (MAT) is considered the *gold standard* for serological diagnosis of leptospirosis, as it can accurately identify the serogroup involved in the infection. However, this test necessitates the use of live strains of *Leptospira* as antigens, making it expensive and requiring a high level of expertise for both execution and interpretation [77]. MAT can be used for detecting anti-*Leptospira* antibodies in the serum of animals across various species, making it applicable to the serological diagnosis of leptospirosis in unconventional companion animals as well. Enzyme-linked immunosorbent assay (ELISA) kits are commercially available for detecting anti-*Leptospira* antibodies, offering an easier and more accessible alternative [77], but they are strictly species-specific, and none are available for unconventional animal species.

*Leptospira* isolation is indeed regarded as the definitive method for confirming the presence of viable leptospires in samples (urine and tissues) from infected animals of any species. However, the requirements for specialized media, advanced equipment, and skilled personnel complicate the process and can contribute to false-negative results. In addition, leptospires typically exhibit slow growth rates, often necessitating several weeks of incubation before successful culture can be established. Therefore, the cultural method is not suitable for prompt diagnosis [79]. Conversely, molecular tests, such as Polymerase Chain Reaction (PCR) and Real-Time PCR, have rapidly become standard diagnostic methods for detecting *Leptospira* in biological samples. Various protocols, each targeting different genes and showing a range of sensitivity and specificity, exist; however, they consistently yield reliable results within a short timeframe [78,79]. Once pathogenic leptospires are detected, it is possible to identify their species and, in some cases, the serovars directly from the DNA extracted from samples [80,81]. Currently, Multilocus Sequence Typing (MLST) stands out as the preferred method of choice for typing *Leptospira*. Three distinct and standardized MLST schemes are available for characterizing isolates, enabling both the identification of species and the inference of the serogroup of isolated strains [82]. Additionally, protocols have been developed to conduct MLST directly on DNA extracted from biological samples, providing a valuable resource for diagnostic and epidemiological applications [83]. However, molecular diagnosis can also fail due to the nature of the analyzed samples. Blood is not always a good specimen for direct diagnosis because bacteremia terminates 7–10 days after infection; urines are negative in the first 10 days post-infection, and when leptospires are localized in kidneys, shedding can be intermittent, particularly in maintenance hosts [77].

## 5. Discussion

The trade and ownership of exotic pets have surged significantly over the past few decades [84]. International agreements exist to regulate the wildlife trade—often bolstered by national or regional laws—with the main focus on the protection of endangered species. Unfortunately, they often overlook crucial factors such as public and animal health implications, as well as the potential impact on biodiversity resulting from the unintentional introduction of invasive species [85].

Owners are usually not sufficiently informed about the risks of contracting infections from their pets. They do not realize that unconventional species may harbor pathogens transmissible to humans, typically associating infections only with traditional species, such as dogs, cats, and birds. Many owners purchase unconventional mammals for their children, under the impression that small and cute animals are easy to care for and suitable as companions.

Moreover, the illegal traffic of pets, in particular those belonging to unconventional species, increases the risk of introducing infected animals into domestic environments. In fact, animals purchased on the black market are not subject to any health checks and may harbor and shed many pathogens, including zoonotic ones. In addition, stress related to breeding and travel conditions makes these animals more susceptible to infections.

Even though purchased animals constitute the majority of unconventional pets, some people keep small wild mammals found outdoors in their homes. The risk of contracting leptospirosis from these animals is high, considering the circulation of leptospires in many areas, mainly where animal species acting as maintenance hosts are present.

Usually, microbiological analyses are not commonly required for clinically healthy animals; therefore, it is difficult to know if the pets are infected and could be potential sources of infection.

Leptospirosis is most commonly associated with dogs, among companion animals, and wild rodents. However, veterinarians do not always remember that animals such as gerbils, degus, gliders, chinchillas, and minipigs are susceptible to *Leptospira*. These animals can often act as maintenance hosts of the spirochaetes and shed leptospires in their urine without showing clinical signs.

In vivo diagnosis of leptospirosis in these animals may also be performed through MAT, using a 1:100 antibody titer as a cut-off, similar to the serological diagnosis in other animal species. The small size of some animal species may discourage the collection of additional blood samples, but MAT could be sufficient to identify infected pets. Diagnosis for leptospirosis in unconventional pets is often only pursued after human cases have been identified. In such instances, the suspected animals are often euthanized, and direct diagnosis for leptospirosis is employed to confirm the source of infection in the owners.

Prophylaxis is possible only through hygienic measures, as no vaccines are available for uncommon mammal pets. Accurate measures to prevent contamination of domestic areas with the urine of infected animals are always crucial. as Additionally, preventive measures should be taken when handling animals and objects such as cages and litter. Beyond that, microbiological analyses to identify *Leptospira* infection in these animals should be conducted, in particular within the first few days after the animals are introduced into the home. Animals kept in breeding facilities and stores often live in groups, which can facilitate the spread of leptospiral infection.

Additional periodic controls are recommended if pets spend part of their time outdoors. In such cases, they can come in contact with the urine of infected wild animals from different species, depending on the environment: rodents and hedgehogs when pets live in private gardens, and small and large mammals when they spend time in rural areas. Moreover, some pets, such as ferrets used in hunting activity, have a high probability of contracting leptospires [65].

The health status of pets of any species always has to be carefully monitored, particularly when immunocompromised individuals live in the household. Special attention is necessary with unconventional companion animals. Pregnant women, the elderly, and individuals with chronic health conditions should avoid direct or indirect contact with these animals. Children, who are highly susceptible to *Leptospira* infection, should also have limited contact with unconventional pets. However, these animals are frequently chosen as gifts for children, including very young ones. The case of leptospirosis in a magician who used his mice for magic tricks in nurseries, where the mice were the source of infection [43], highlights the superficiality with which these animals often are kept.

## 6. Conclusions

New pets are increasingly present in domestic environments and in contact with owners, who are commonly unaware of the risk they represent. Leptospirosis is a zoonotic infectious disease well known in domestic animals such as dogs, whereas it is not commonly associated with unconventional mammal pets. This limitation is also related to the paucity of studies and data about the epidemiological and pathological characteristics of *Leptospira* infection in these new pets. For these reasons, and considering the importance of leptospirosis from a One Health perspective, studies on this topic are necessary, and specific diagnostic measures to identify infected companion animals early are pivotal.

## Figures and Tables

**Table 1:** Documented cases of human leptospirosis associated with unconventional mammal pets.

Human Case	Etiologic Agent	Pets Source of Infection	Provenience of Pets	Country	Reference
Man	*L. interrogans* sv Icterohaemorrhagiae	Rats	Pet shop	UK	[37]
Man	*L. interrogans* sv Icterohaemorrhagiae/ Canicola	Rats	Pet shop	Germany	[38]
Woman	*L. interrogans* sv Icterohaemorrhagiae	Rat	Wild environment	UK	[39]
Man	*L. interrogans* sv Icterohaemorrhagiae/ Copenhageni	White rats	Pet shop	Sweden	[40]
Woman	*L. borgpeterseni* sv Sejroe	Mouse	Pet shop	Denmark	[41]
Woman	*L. borgpeterseni* sg Sejroe	Mice	Pet shop	France	[43]
Man *	*L. borgpeterseni* sg Ballum/*L. interrogans* sg Pyrogenes	Mouse	Pet shop	Belgium	[43]
Woman *	*L. borgpeterseni* sg Ballum/*L. interrogans* sg Pyrogenes	Mouse	Pet shop	Belgium	[43]
Woman	*L. interrogans* sg Icterohaemorrhagiae	Rats	Pet shop	Belgium	[43]
Man	*L. interrogans* sg Icterohaemorrhagiae	Rat	Pet shop	France	[43]
Woman	*L. interrogans* sg Icterohaemorrhagiae	Rat	Pet shop	France	[43]
Woman	*Leptospira* spp.	Sugar glider	Pet shop	Rhode Island	[57]
Man	*L. interrogans*	Ferrets	Bred for hunting activity	France	[65]

Legend: * same source of infection; sv: serovar; sg: serogroup.

**Table 2:** Correlation between unconventional mammal pets and *Leptospira*.

Order	Family	Genus	Species	Common Name	Susceptibility to Infection	Epidemiological Role	Verified *Leptospira* Infection in Pets
Rodentia	Cricetidae	*Mesocricetus*	*M. auratus*	Golden hamster or Syrian hamster	Documented	Incidental host	Yes
		*Phodopus*	*P. campbelli*	Campbell’s dwarf Russian hamster	Suspected	Unknown	No
			*P. roborovski*	Roborovski dwarf hamster	Suspected	Unknown	No
			*P. sungorus*	Dwarf winter white Russian hamster	Suspected	Unknown	No
		*Cricetus*	*C. cricetus*	European hamster	Suspected	Unknown	No
			*C. griseus*	Chinese hamster	Suspected	Unknown	No
	Caviidae	*Cavia*	*C. porcellus*	Young guinea pigs	Yes	Incidental and maintenance host	Yes
	Muridae	*Rattus*	*Rattus* spp.	Rat	Yes	Maintenance host	Yes
		*Mus*	*Mus* spp.	Mouse	Yes	Maintenance host	Yes
		*Meriones*	*Meriones* spp.	Gerbil	Yes	Incidental host (mainly)	No
	Octodontidae	*Octodon*	*O. degus*	Degu	Yes	Maintenance host	No
	Chinchillidae	*Chinchilla*	*C. lanigera*	Long-tailed chinchilla	Yes	Incidental host	No
Diprotodontia	Petauridae	*Petaurus*	*P. breviceps*	Sugar glider	Suspected	Unknown	No
Eulipotyphla	Erinaceidae	*Erinaceus*	*E. europaeus*	European hedgehog	Yes	Maintenance host	No
		*Atelerix*	*A. albiventris*	African pygmy hedgehog	Yes	Maintenance host	No
Carnivora	Mustelidae	*Mustela*	*M. putorius furo*	Ferret	Yes	Maintenance host (probably)	Yes
Lagomorpha	Leporidae	*Oryctolagus*	*O. cuniculus*	European rabbit	Yes	Maintenance host	Yes
Artiodactyla	Suidae	*Sus*	*S. scrofa domesticus*	Minipigs or miniature pigs	Yes	Maintenance and incidental host	No

## Data Availability

Not applicable.
